# Preputial cyst in the newborn: a case report

**DOI:** 10.11604/pamj.2026.53.155.50885

**Published:** 2026-04-14

**Authors:** Kamal El Haissoufi, Amine El Wardi

**Affiliations:** 1 Uro-Visceral and Genital Pediatric Surgery, Moulay El Hassan Ben El Mehdi Hospital, Laayoune, Morocco,; 2 Annasr Laboratory of Pathology, Laayoune, Morocco

**Keywords:** Preputial cyst, median raphe cyst, surgery, child, case report

## Abstract

Preputial cysts are rarely seen in the pediatric population, with most reported cases from Asia. This condition falls within the category of median raphe cysts, which encompasses various possible locations from urethral meatus to the anus. The diagnosis remains clinical and must be confirmed by histology. Surgical excision is still the treatment of choice with a negligible risk of complications and recurrence. Here, we report the case of a newborn who was successfully treated by circumcision for a preputial cyst diagnosed immediately after birth. Given that the lesion can occur many years after birth, the universal practice of circumcision early in life in most countries of Africa may explain its rarity in our settings.

## Introduction

Preputial cyst is a rarely reported condition with most documented cases from Asia [1]. Notably, only a few cases from Africa were described, including our case [2]. Various doctors, including surgeons, pediatricians, and dermatologists, may encounter the disease in patients of varying ages [1]. Also, different therapeutic options were practiced in managing patients [3]. Here, we report the case of a preputial cyst in a newborn treated after birth in our center. Hence, we discuss clinical aspects, diagnosis, treatment modalities, and evolution features.

## Patient and observation

**Patient information:** a 3.1 kg male newborn was born by a normal vaginal delivery at 38-week gestational age in our hospital. No significant pathological history in the family nor during pregnancy was reported. Also, no genetic predisposition to any diseases was confirmed by the parents.

**Figure 1:**
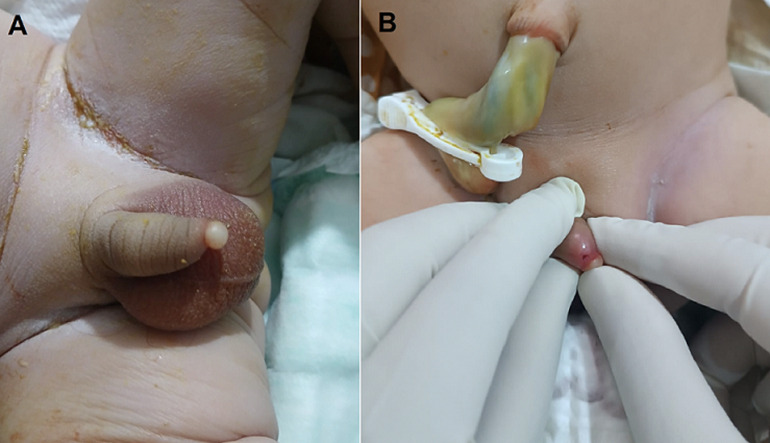
A,B) clinical examination of the genitalia after birth showing a complete and circumferential foreskin with a 6 mm cystic lesion located distally and ventrally making its retraction difficult

**Clinical findings:** our intervention was directly sought-after birth because of a noticed abnormality of the phallus by delivery nurses and the pediatrician. Clinical examination found an apyretic newborn in good general condition without any associated malformation. On genital examination, both testicles were palpable and in a normal intrascrotal position. Penile size was within normal range with a normal apical urethral meatus. The foreskin was complete and circumferential, not easily retractable because of a 6 mm cystic lesion located ventrally and distally arising from the median raphe (Figure 1).

**Timeline of current episode:** clinical assessment on the first day of life. Ultrasound and biological tests were performed after clinical examination. Surgical intervention was performed on the 2^nd^ day of life. Findings of histological analysis were obtained one week afterwards.

**Diagnostic assessment:** a reno-vesical ultrasound was performed and showed no signs of urinary tract obstruction or renal parenchymal abnormality. All serum parameters were also within normal range. No diagnostic challenges in our patient could be reported.

**Figure 2:**
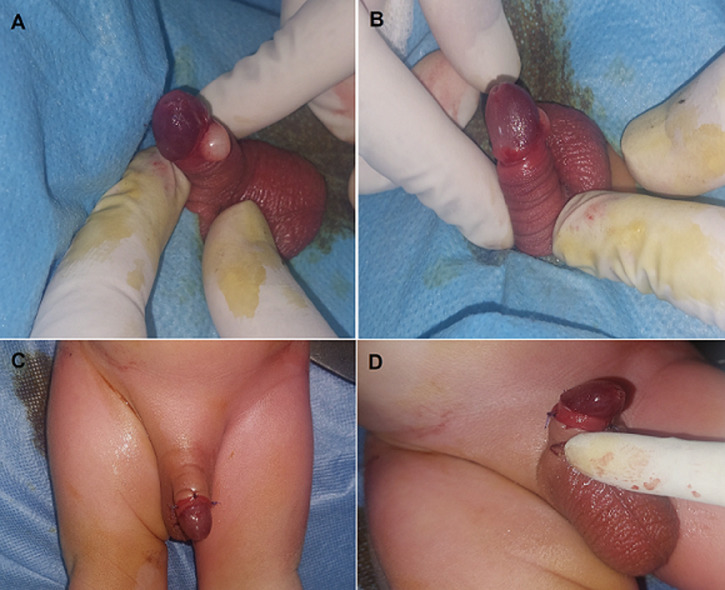
A,B) perioperative views after foreskin retraction showing normal glans and urethral meatus with a cystic lesion arising from the median raphe; C,D) final aspect after performed circumcision with excision of the lesion

**Therapeutic interventions:** the newborn was admitted to the operating room on the 2^nd^ day of life, where a classical circumcision with removal of the cyst was performed. The intervention was done under a dorsal penile block by 1% xylocaine (Figure 2). The whole surgical intervention was performed by a senior pediatric surgeon with the aid of two operating room assistants. The postoperative time was uneventful with no hemorrhagic or infectious complications.

**Figure 3:**
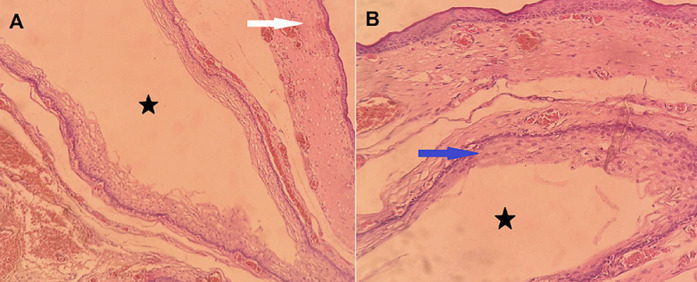
A,B) histopathology of the sample showing a cystic cavity (black stars), lined by a squamous epithelium (blue arrow), and covered with skin and subcutaneous tissue (white arrow) (H and E, 10X and 40X)

**Diagnosis:** histological analysis of the specimen showed a cystic cavity, lined by a squamous epithelium and covered with skin and subcutaneous tissue (Figure 3).

**Follow-up and outcome of interventions:** a good wound healing postoperatively after 10 days was observed, and the parents were totally satisfied with the cosmetic result after four months of follow-up. Also, no negative incidents in terms of adherence and tolerability were observed. Taken together, the evolution of the disease in our patient, our management, and follow-up are summarized in the timeline of Table 1.

**Table 1:** a timeline showing the evolution of the disease in our patient, its medical management and follow-up

Before birth	At birth	Surgical consultation	Postsurgical management	After four months of follow-up
Normal pregnancy monitoring	Abnormality of the phallus on standard newborn examination	-Normal physical appearance; -Normal reno-vesical ultrasound and serum parameters; -Circumcision; -Histological diagnosis	Local wound care	-Good wound healing; -Good cosmetic result

**Patient´s perspective:** “I have an expectation to see our child without any signs of cyst recurrence. Also, we are totally satisfied with the results, and we remain available to continue the medical follow-up process.”

**Informed consent:** written informed consent was obtained from the patient´s parents for publication of this case report and accompanying images.

## Discussion

Preputial cysts represent a rare condition where fewer than 200 cases are reported in the literature, and our case is only the second reported one from Africa [2]. This condition was first described by Mermet P in 1895 as a “congenital cyst of the genitoperineal raphe” [4]. Almost 70 years ago, Thompson LM and Lantin PM reported two cases of parameatal cysts of the glans penis in adults, although the disease was present since birth, considering it a rarely encountered congenital benign cyst of the penis [5]. Embryologically and during the process of preputial delamination from the glans, cystic spaces appear normally at the ventral area and then must be obliterated throughout the progression around the 8^th^ month of pregnancy. However, the persistence of these cystic areas might be the origin of the disease near the ventral surface of the urethral meatus [5]. Therefore, since these cysts can occur from the urethral meatus to the anus, median raphe cysts (MRCs) are logically attributed to the disease, which encompasses all possible locations [6]. Indeed, various terms were previously used to describe the lesion, including parameatal cyst, mucus or mucoid cyst of the penis, urethroid cyst, median raphe cyst of the penis, cysts of the genito-perineal raphe, apocrine cystadenoma, and apocrine hidrocystomas, which can all be considered synonymous [6].

Mostly, the lesion is present since birth but can occur afterwards at any age, including adulthood [1]. Mainly, the cyst is solitary, and its size can vary from 1 mm to more than 3 cm. The disease is usually asymptomatic unless complicated by infection or trauma. Also, difficulties with sexual intercourse and psychological embarrassment can lead the patient to the surgeon in adulthood. The diagnosis remains clinical and must be confirmed by histology since radiological investigations present a limited role. Importantly, the differential diagnosis differs depending on the location of the cyst [1]. Globally, MRCs can be differentiated from urethral diverticulum, glomus tumor, dermoid and epidermoid cysts, pilonidal cyst, and steatocystoma [7]. Treatment options include simple observation, aspiration of the cyst, marsupialization or unroofing, excision with primary closure, or circumcision in the presence of a preputial cyst [1]. Expectant treatment can be reasonably adopted, especially in front of small cysts, considering that most cases remain asymptomatic, and a spontaneous resolution is possible during the first two years of life [8,9]. Aspiration of the cyst is associated with a considerable risk of recurrence [3]. Also, unroofing or marsupialization should be avoided because of secondary gaping sinus development, responsible for an unsatisfying cosmetic result [10]. Consequently, complete excision of the cyst is widely considered the most efficient, cosmetic, and the least likely to be associated with subsequent recurrence [1,9,10]. An uneventful course without penile pain, voiding symptoms, or cosmetic dissatisfaction is the rule in children after surgical excision [9]. However, urethrocutaneous fistula can exceptionally occur following surgical excision [3]. Immediately after birth, the diagnosis in our patient was made clinically and confirmed thereafter by histology. Even though the cyst was small and symptom-free, circumcision was the treatment of choice considering the preputial location of the cyst and the request of the parents, as it is routinely performed in our context because of cultural and religious reasons.

## Conclusion

Through this paper, we present a case of a preputial cyst diagnosed after birth and treated surgically with a good evolution. The diagnosis is first clinical and must be confirmed by histology. Surgical excision is the treatment of choice with a very low risk of recurrence and complications. The paucity of similar cases reported in Africa may be related to the routine circumcision for children in many countries.
